# To Evaluate & Compare Retention of Complete Cast Crown in Natural Teeth Using Different Auxiliary Retentive Features with Two Different Crown Heights - An *In Vitro* Study

**Published:** 2015-06

**Authors:** Kundapur Vinaya, Hegde Rakshith, Krishna Prasad D., Shetty Manoj, Mankar Sunil, Shetty Naresh

**Affiliations:** 1 Senior Lecturer, Department of Prosthodontics, M R Ambedkar Dental College & Hospital, Bangalore, Karnataka India;; 2 Professor, Department of Prosthodontics, A. B. Shetty Memorial Institute of Dental Sciences, Deralakatte, Mangalore, Karnataka, India;; 3 Reader, Department of Conservative dentistry & Endodontics, DA Pandu Memorial-RV Dental College & Hospital, Bangalore, Karnataka, India;; 4 Reader, Department of Prosthodontics, AL-Azhar Dental College, Thodupuzha, Kerala, India

**Keywords:** retention, tensional forces, proximal grooves, proximal boxes, castings

## Abstract

**Background & objectives:**

To evaluate the retention of complete cast crowns in teeth with adequate and inadequate crown height and to evaluate the effects of auxiliary retentive features on retention form complete cast crowns.

**Materials and methods:**

Sixty freshly extracted human premolars. They were divided into 2 major groups depending upon the height of the teeth after the preparation. Group1 (H1): prepared teeth with constant height of 3.5 mm and Group 2 (H2): prepared teeth with constant height of 2.5 mm. Each group is further subdivided into 3 subgroups, depending upon the retentive features incorporated. First sub group were prepared conventionally, second sub group with proximal grooves and third subgroups with proximal boxes preparation. Castings produced in Nickel chromium alloy were cemented with glass ionomer cement and the cemented castings were subjected to tensional forces required to dislodge each cemented casting from its preparation and used for comparison of retentive quality. The data obtained were statistically analyzed using Oneway ANOVA test.

**Results:**

The results showed there was statistically significant difference between adequate (H1) and inadequate (H2) group and increase in retention when there was incorporation of retentive features compared to conventional preparations. Incorporation of retentive grooves was statistically significant compared to retention obtained by boxes. Results also showed there was no statistically significant difference between long conventional and short groove.

**Conclusion:**

Complete cast crowns on teeth with adequate crown height exhibited greater retention than with inadequate crown height. Proximal grooves provided greater amount of retention when compared with proximal boxes.

## INTRODUCTION

A plethora of restorations are used to restore form and functions of teeth which include intracoronal restorations, extracoronal restorations, fixed dental prosthesis, and removable dental prosthesis and implant supported prosthesis. Though implant supported prosthesis revolutionized field of dentistry for replacement of missing teeth, fixed dental prosthesis are still the most frequent restorative procedure. Tooth preparation is a far most important phase of treatment it must be done with skill and meticulous attention to detail, for everything that follows good esthetic, proper occlusion, protection of remaining tooth structure and longevity of restoration. It becomes increasingly important to understand functions of retention in cemented tooth castings. Restorations should meet its functional, biological and esthetic requirement with its capability of retention and resistance, Retentive enough to resist removal of restoration along the path of insertion under this condition cement bond is subjected to tension and shear and should have Resistance so as to prevent dislodgement of restoration by forces directed in apical, oblique or horizontal direction (1).

The purpose of the study is to compare the retention of complete cast crown with adequate & inadequate crown heights and to compare the effect of auxiliary retentive features (proximal grooves, proximal boxes) on retention of complete cast crowns.

## REVIEW OF LITERATURE

Rules of retention as proposed by various studies depends on various factors like height of prepared tooth, surface area, texture of the surface, degree of convergence of opposing walls of preparation and retentive features of the preparation.

Kaufman *et al* (2) studied the factors effecting retention pertaining to three categories based on retention in prepared tooth based on tooth surface, height and texture, Retention in casting with relative adaptation of casting to tooth surface, retention that are functions of cementing medium. They concluded that increased retention with increase in height and diameter, in convergent preparation area closer to gingival termination contributed to greater retention and also each unit area has comparable retentive ability regardless of other dimensions of preparation.

Tijan *et al* (3) determined the biometric groove placement on three quarter crowns based on biologic, functional and structural requirement and found that maximum structural reinforcement of buccal veneering margin, the two grooves must be continuously connected to each other by means of occlusal offset. However if the curvature of tooth sometimes prevents this geometry attempt should be made for this continuity between grooves and offset so as to create excessive stress concentration at their junction.

Kishimoto (4) investigated the influence of preparation features on retention and resistance of three quarter crowns it was found that retention increases with groove placement in lingual placement in lingual position and greatest retention was seen with multiple grooves.

Owen (5) stated that proximal boxes give superior results over grooves and also stated grooves increase resistance not retention in complete coverage restorations.

Omar (6) compared the retentive capacity of zinc phosphate, zinc polycarboxylate and glass ionomer cemented and concluded that glass ionomer cement were distinctly superior to retain complete coverage castings. But, Mowafy (7) determined the retention of metal ceramic crowns cemented with resin cements were better than glass inonomer cement.

Pai *et al* (8) evaluated and compared the retention of complete cast restoration with two different crown heights with incorporation of retentive features and concluded that retention increased with adequate crown height and with proximal grooves.

## MATERIALS AND METHODS

**Figure 1:**
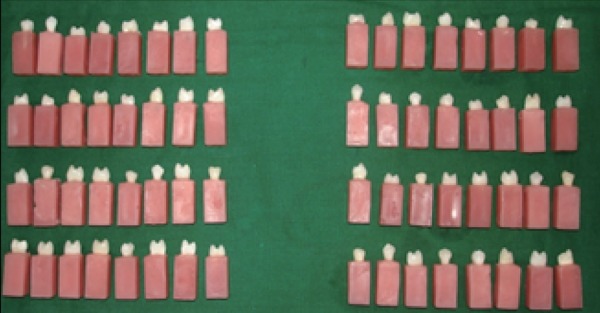
The teeth are mounted vertically in auto polymerizing acrylic resin

60 maxillary first premolars with good coronal anatomy, which are extracted for orthodontic purposes, are selected. After the extraction of teeth are disinfected with 5.2% sodium hypochlorite & stored in normal saline. The roots of all teeth are notched for anchorage and mounted vertically in autopolymerizing acrylic resin (Fig. 1). Tooth mounted in autopolymerizing acrylic resin is checked for parallelism of axial walls using a surveyor. A high speed air turbine hand piece (Panamax, NSK) was mounted to the vertical arm of surveyor using a custom made jig, thus making at as a custom made paralleling milling device. The airotor hand piece was used with water spray to prevent desiccation of dentine.

**Figure 2:**
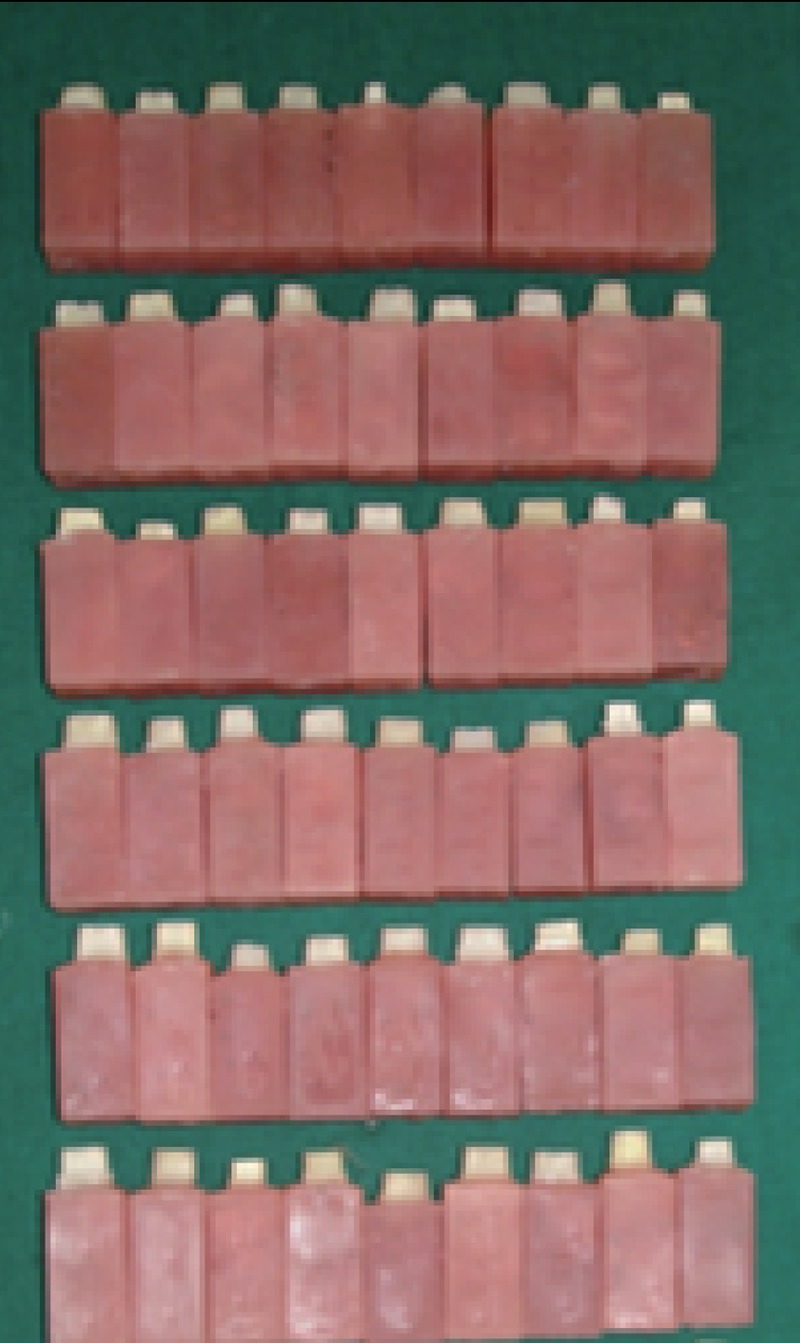
Prepared teeth mounted vertically in auto polymerizing acrylic resin

The occlusal surface was sectioned flat with the help of small wheel diamond bur. Axial reduction was done by using round end tapering fissure diamond bur (102 R Shofu).The bur is held vertically to prepare the axial walls. The divergence of the bur gives the uniform convergence of the teeth of about 6-10 degrees. Approximately 1-1.5 mm of axial tooth structure was removed by preparation. All the preparation terminated in the dentine (Fig. 2).

**Figure 3:**
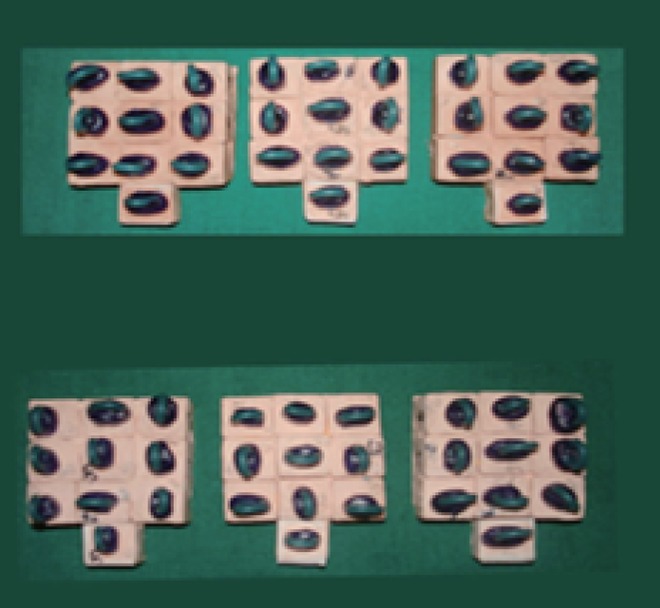
Sprued wax patterns

**Figure 4:**
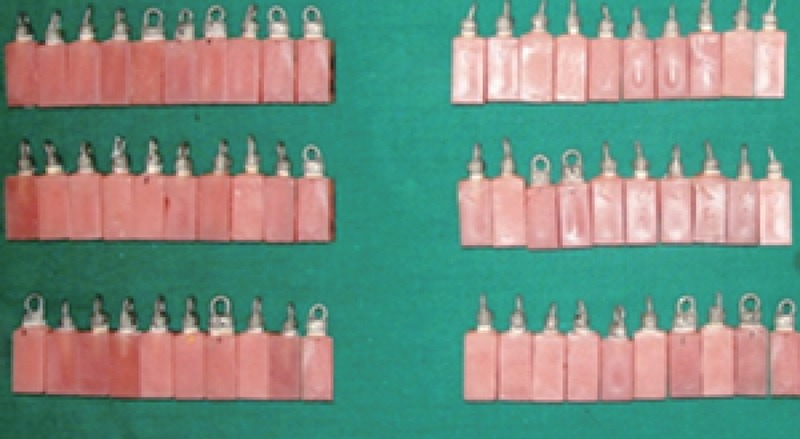
Completed castings verified on preparation

The 60 teeth are divided into 2 major groups depending upon the height of the teeth after preparation. Group 1 (H1) consisted of 30 prepared teeth with constant Height of 3.5 mm and Group 2 (H2) consisted of 30 prepared teeth with constant Height of 2.5 mm. Each group was further subdivided into 3 subgroups, depending upon the retentive features to be incorporated. Group I with conventional preparation (AH1), proximal grooves (BH1), proximal boxes (CH1) consisted of 10 prepared teeth for each subgroup. Group II with conventional preparation (AH2), proximal grooves (BH2), and proximal boxes (CH2) consisting of 10 prepared teeth in each subgroup. Proximal grooves are placed using diamond points.Two grooves are placed each in centre of mesial and distal surface and terminated 0.5 mm above the finish line. Grooves are placed parallel to path of insertion. Depth of the groove equals half the diameter of diamond points. Using diamond bur two boxes are placed each in the centre of mesial and distal surface. Depth of the box 0.5 mm and width 2 mm gingival wall of the box extended 0.5 mm above the chamfer line. Impressions of all the samples in each group were made using polyvinylsiloxane impression material (3M ESPE, Express ^TM^ XT Putty soft) and polyvinylsiloxane impression material light body consistency with single step double mix technique. Prepared tooth mounted on acrylic block was removed from impression and verified for any defects. Impression was cleaned and dried and poured with die stone (Type IV gypsum, Kalrock).The set model was carefully examined for any defects or air bubbles. The die spacer (Pico–Fit) was applied on the occlusal and axial surface of the die, excluding band of 1mm immediately adjacent to finish line. Die lubricant (Iso-Lite) was painted on the die the wax copings of approximately 1mm were prepared using blue inlay wax carved with PKT instrument simulating a cylindrical axially and flat occlusally (Fig. 3). A 2 mm sprue wax was made as loop and attached to the centre of occlusal surface of wax pattern for testing universal testing machine. The wax patterns was sprued and invested with phosphate bonded investment. Manufacturer’s instructions were strictly followed during the investing procedure. Burn out was done by lost wax process. The test copings were casted using the nickel chromium alloy (wiron 99, Bego, Germany) in electric centrifugal induction casting machine. The surface of the castings was air abraded with 100 µ aluminum oxide particles. Then the fit of the completed castings were verified on preparation (Fig. 4).

**Figure 5:**
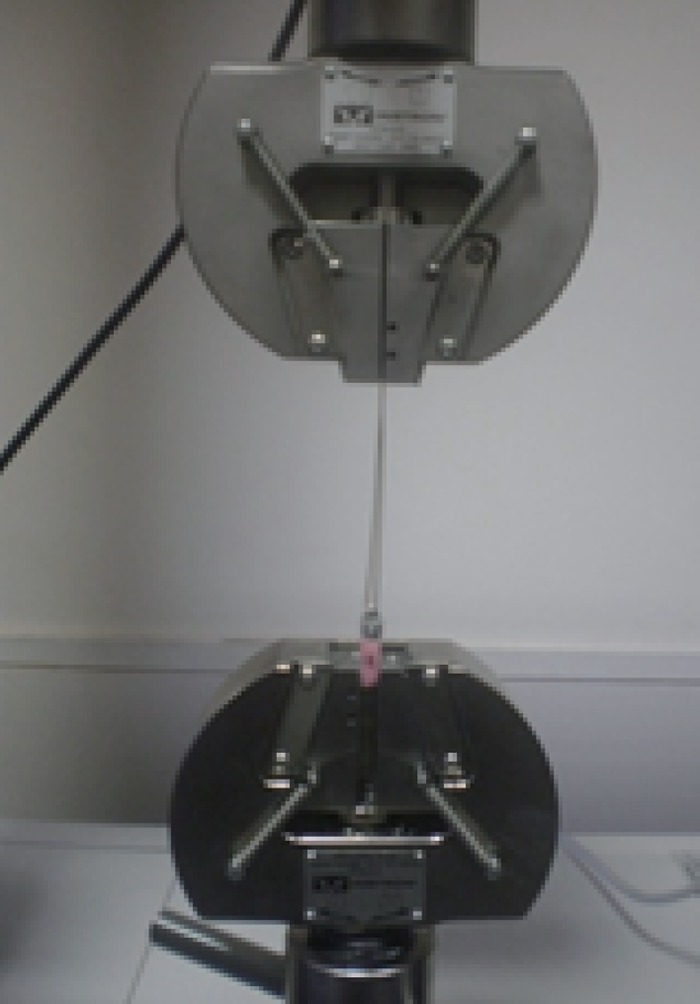
Castings on testing machine

All the samples in the group A, B, C, AH1, BH1, CH1, AH2, BH2 and CH2 were cemented with glass ionomer cement (GC Gold label) respectively. All cementation were done on the same day by single operator using firm finger pressure. All the test specimens were stored for 24 hours at 37°C at 100% relative humidity before mechanical testing. The crowns were subjected to a vertical dislodgement force until failure on a universal testing machine (INSTRON) at a cross head speed of 0.5 mm/min. The castings were pulled along the apico coronal axis of each tooth using a “J” hook attached to the upper member of the testing machine (Fig. 5). The force at dislodgement and debonding were recorded in Newton’s and tabulated.

## STATISTICAL ANALYSIS AND RESULTS

Statistical analysis was carried out using SPPSS 11.5 version. ANOVA with post hoc test (Tamhane’sT2) was applied for inter and intra group comparison. The mean and standard deviation of the forces required to dislodge castings is shown in the Table 1, Intergroup comparison of forces between the group is shown is Table 2 & Table 3 shows intra group comparison of forces required to dislodge the castings. The results showed there was statistically significant difference between adequate (H1) and inadequate (H2) group. Within the group comparison showed there was statistical significance difference between long conventional and long groove. Long groove showed statistically significant difference between long boxes, short conventional, short groove and short box .Long box preparation showed statistically significance to short conventional, short groove and short box. Short conventional showed statistical significance in relation to short groove and short box, short groove and short box were also statistically significant.

**Table 1:** Descriptive statistics of forces (N) required to dislodge the castings from tooth preparation

	SUB GROUPS	MEAN ± S.D

Long (3.5 mm)	Conventional preparation	19.53 ± 2.99
	Groove preparation	316.96 ± 38.71
	Box preparation	141.73 ± 26.91
Short (2.5 mm)	Conventional preparation	11.01 ± 2.29
	Groove preparation	20.08 ± 1.24
	Box preparation	15.24 ± 0.71

**Table 2:** Inter group comparison of the forces between the groups

ANOVA VAR00003					
	Sum of Squares	df	Mean Square	F	Sig.

Between Groups	757707.415	5	151541.483	405.779	0.000
Within Groups	20166.739	54	373.458		
Total	777874.154	59			

**Table 3:** Intra group comparison of the forces required to dislodge cast

VAR00003 Tamhane						
(I) group	(J) group	Mean Difference (I-J)	Std. Error	Sig.	95% Confidence Interval	
					Lower Bound	Upper Bound

long control	long groove	-297.43091^a^	12.27725	0.000	-345.6148	-249.2470
	long box	-122.18991^a^	8.55860	0.000	-155.6561	-88.7238
	short con	8.52109^a^	1.15914	0.000	4.6313	12.4108
	short groove	-.54691	.98550	1.000	-4.0322	2.9384
	short box	4.29465^a^	.93384	0.011	.8543	7.7350
long groove	long control	297.43091^a^	12.27725	0.000	249.2470	345.6148
	long box	175.24100^a^	14.91138	0.000	124.0772	226.4048
	short con	305.95200^a^	12.26553	0.000	257.7546	354.1494
	short groove	296.88400^a^	12.25034	0.000	248.6687	345.0993
	short box	301.72556^a^	12.24630	0.000	253.5054	349.9457
long box	long control	122.18991^a^	8.55860	0.000	88.7238	155.6561
	long groove	-175.24100^a^	14.91138	0.000	-226.4048	-124.0772
	short con	130.71100^a^	8.54178	0.000	97.2269	164.1951
	short groove	121.64300^a^	8.51995	0.000	88.1341	155.1519
	short box	126.48456^a^	8.51413	0.000	92.9687	160.0004
short con	long control	-8.52109^a^	1.15914	0.000	-12.4108	-4.6313
	long groove	-305.95200^a^	12.26553	0.000	-354.1494	-257.7546
	long box	-130.71100^a^	8.54178	0.000	-164.1951	-97.2269
	short groove	-9.06800^a^	.82676	0.000	-11.9812	-6.1548
	short box	-4.22644^a^	.76445	0.003	-7.0738	-1.3791
short groove	long control	.54691	.98550	1.000	-2.9384	4.0322
	long groove	-296.88400^a^	12.25034	0.000	-345.0993	-248.6687
	long box	-121.64300^a^	8.51995	0.000	-155.1519	-88.1341
	short con	9.06800^a^	.82676	0.000	6.1548	11.9812
	short box	4.84156^a^	.46043	0.000	3.2341	6.4491
short box	long control	-4.29465^a^	.93384	0.011	-7.7350	-.8543
	long groove	-301.72556^a^	12.24630	0.000	-349.9457	-253.5054
	long box	-126.48456^a^	8.51413	0.000	-160.0004	-92.9687
	short con	4.22644^a^	.76445	0.003	1.3791	7.0738
	short groove	-4.84156^a^	.46043	0.000	-6.4491	-3.2341

a The mean difference is significant at the 0.05 level.

**Figure 6:**
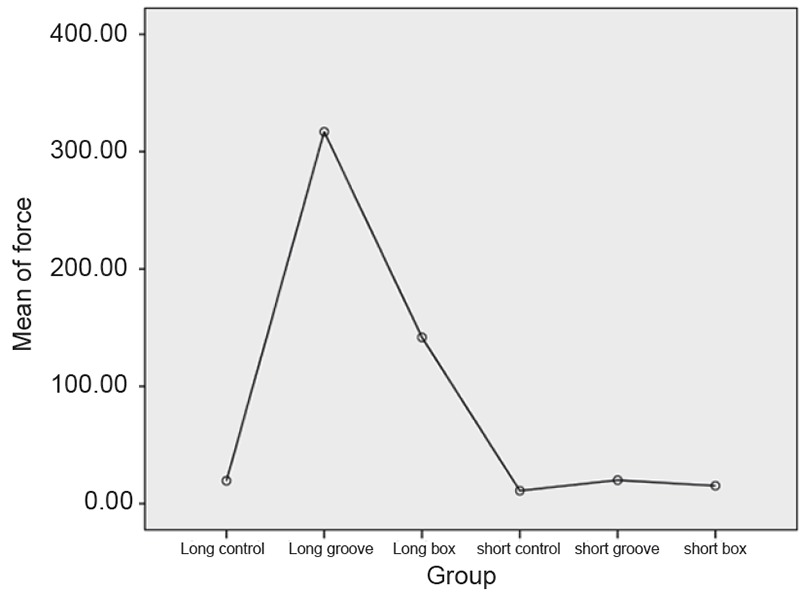
Showing mean force required to dislodge the castings in different groups.

Within the limitation of the study the results showed that there was statistically significant difference between the adequate and inadequate group. The results also showed there was increase in retention when there was incorporation of retentive features compared to conventional preparations. Incorporation of retentive groove was statistically significant compared to retention obtained by box. Results also showed there was no statistically significant difference between long conventional and short groove (Fig. 6).

## DISCUSSION

Retention is considered to be the prevention of removal of cast restoration along its path of insertion; résistance can be defined as preventing dislodgement by apical / oblique forces and preventing any movement of restoration under occlusal forces (3). The maximum amount of force required to dislodge the castings cemented on the prepared tooth with complete cast crown was studied here by considering different auxiliary retentive features and two different crown heights in the present study. Cemented castings were subjected to tensile strength testing apparatus. The results in the present study showed statistically highly significant increase in retention of crowns on tooth with adequate crown (H1) compared to inadequate crown height (H2), this attributed to the fact pointed by Kaufman *et al* as increased retention of complete cast crown depends on length, surface area, surface texture, convergence angle and other related factors to castings and cementations (2).

Similarly the results also showed there was increase in retention when there was incorporation of retentive features compared to conventional preparations. Incorporation of retentive grooves was statistically significant compared to retention obtained by boxes. Results also showed there was no statistically significant difference between long conventional and short groove. However in a study conducted by Pai *et al* (8) the crown with adequate crown height provided better retention compared to inadequate crown height but boxes provided better retention compared to grooves and conventional preparation.

In the present study the retentive grooves were incorporated into the tooth on proximal surface during the preparation so there was no difficulty encountered to choose the site for placement of groove, the cast restoration enhancing retentive qualities for the cemented restoration. Chan and Boyer (9) in their study proposed the concept of auxiliary retention by placing opposing grooves in the castings and the cavity perpendicular to path of withdrawal and extending around the entire circumference of pattern midway between the surface and the base. After cementation grooves were occupied with cement. To dislodge the casting fracture of cement or the dentine must occur. Here in his study the retentive grooves were incorporated in the prepared tooth before cementation according to the groove provided on the restoration the draw back was need for the proper care while placing the groove exactly coinciding with the cast restoration there was possible chance of weakening the prepared teeth.

Craig *et al* (10) study reported that deep developmental groove carved near the center of tooth should be avoided because they will tend to produce deleterious stress concentration. He also suggested that high compressive stress on interior surface of crown may be avoided by rounding reduced cusps. In the present study proximal grooves were considered in a standardized manner as grooves were placed using diamond points .Two grooves were placed each in centre of mesial and distal surface and terminated 0.5 mm above the finish line .Grooves were placed parallel to path of insertion .Depth of the groove equals half the diameter of diamond points was maintained.

Tylman (11) suggests placing grooves just buccal to junction of buccal and middle third of proximal surface of tooth provides retention. Grooves gave insignificant gain in retention in a study conducted by Potts *et al* (12) which reported that grooves and coverage of distal half of buccal surface produced a cumulative effect on résistance, grooves produced small increase in retention marked increase in resistance. Grooves on proximal surface provided complete resistance to horizontal dislodgement compared with grooves on buccal and lingual surface which provided partial resistance (1). In the present study grooves were provided on the mesial and distal surface and they provided better retention compare to retentive boxes and conventional preparations.

According to the present study occlusal and axial reduction alone gives insufficient retention in comparison with addition of proximal grooves and boxes. Adequate crown height and grooves gave cumulative effect on retention of complete cast crowns. An overview on the results in this present study showed that occlusogingival length plays major factor in providing retention. Proximal groove are conservative and provide better auxiliary retentive feature when compared to boxes. This finding is valuable for teeth with short clinical height. However in case where abutment tooth has caries or fractured restorations utilization of boxes will aid in retention.

In the present study care was taken to maintain proper taper and height during the tooth preparation by using custom made paralleling device using surveyor. Impressions of the prepared tooth were made using single step double mix impression technique using putty and light body polyvinylsiloxane impression material. Manufacturer’s instructions were strictly followed during the cementation procedure. Similar protocols were followed in a study conducted by Pai et al where proximal boxes provided better retention compared to incorporation of proximal grooves. Sample size was doubled compared to the above mentioned study to ensure validity of statistical analysis.

Further clinical studies are required on this topic to verify the effect of auxiliary retentive features on retention of complete cast crowns in teeth with adequate and inadequate crown height. Further more there are various factors which affect the retention of complete crowns, incorporation of grooves cannot be solely considered as major factor.

## SUMMARY

The most common cause for failure of full coverage restoration is loss of retention. On the basis of clinical experience factors and properties which seem to pertain to and affect retention have been recorded. Thus retention is a function of taper, height, surface area and texture of tooth preparation.

This study was performed to evaluate retention of complete cast crown with adequate and inadequate crown height and also comparative evaluation of effect of auxiliary retentive features such as proximal grooves and boxes was done. In this study 60 freshly extracted human premolar teeth were mounted on acrylic blocks. The mounted teeth were prepared to receive complete cast crown using a custom made paralleling device to standardize the axial inclination. They were grouped as long and short based on 3.5 mm and 2.5 mm crown height and sub grouped as conventional, proximal groove and proximal box respectively. Samples were cemented with glass ionomer cement. Cemented samples were tested using universal testing machine until failure and axial dislodgement force were calculated.

Within the limitation of the study the results showed that there was statistically significant difference between the adequate crown height group and inadequate crown height group. The results also showed there was increase in retention when there is incorporation of retentive features compared to conventional preparations. Incorporation of retentive grooves showed better retention compared to retention obtained by boxes. Results also showed there was no statistically significant difference between long conventional and short groove.

## CONCLUSIONS

From this study following conclusions can be drawn:

1. Complete cast crowns on teeth with adequate crown height exhibited greater retention than with inadequate crown height.
2. Increased retention was seen by incorporation of auxiliary retentive features compared to conventional preparations.
3. Proximal grooves provided greater amount of retention when compared with proximal boxes.
4. Incorporation of proximal groove in inadequate crown provided equal retention as conventional preparation with adequate crown height.
